# DNA damaging agent-induced apoptosis is regulated by MCL-1 phosphorylation and degradation mediated by the Noxa/MCL-1/CDK2 complex

**DOI:** 10.18632/oncotarget.9217

**Published:** 2016-05-07

**Authors:** Wataru Nakajima, Kanika Sharma, June Young Lee, Nicolas T. Maxim, Mark A. Hicks, Thien-Trang Vu, Angela Luu, W. Andrew Yeudall, Nobuyuki Tanaka, Hisashi Harada

**Affiliations:** ^1^ Phillips Institute for Oral Health Research, School of Dentistry, Massey Cancer Center, Virginia Commonwealth University, Richmond, Virginia, USA; ^2^ Department of Molecular Oncology, Institute for Advanced Medical Sciences, Nippon Medical School, Kawasaki, Japan; ^3^ Department of Oral Biology, Dental College of Georgia, Augusta University, Augusta, Georgia, USA

**Keywords:** CDK2, MCL-1, Noxa, phosphorylation, chemotherapy

## Abstract

Noxa, a BH3-only pro-apoptotic BCL-2 family protein, causes apoptosis by specifically interacting with the anti-apoptotic protein MCL-1 to induce its proteasome-mediated degradation. We show here that the DNA damaging agents cisplatin and etoposide upregulate Noxa expression, which is required for the phosphorylation of MCL-1 at Ser64/Thr70 sites, proteasome-dependent degradation, and apoptosis. Noxa-induced MCL-1 phosphorylation at these sites occurs at the mitochondria and is primarily regulated by CDK2. MCL-1 and CDK2 form a stable complex and Noxa binds to this complex to facilitate the phosphorylation of MCL-1. When Ser64 and Thr70 of MCL-1 are substituted with alanine, the mutated MCL-1 is neither phosphorylated nor ubiquitinated, and becomes more stable than the wild-type protein. As a consequence, this mutant can inhibit apoptosis induced by Noxa overexpression or cisplatin treatment. These results indicate that Noxa-mediated MCL-1 phosphorylation followed by MCL-1 degradation is critical for apoptosis induced by DNA damaging agents through regulation of the Noxa/MCL-1/CDK2 complex.

## INTRODUCTION

DNA damaging agents, such as cisplatin and etoposide, are employed for the treatment of a wide array of solid tumors, but the prolonged use of chemotherapeutic drugs is limited by their toxicity and by the development of resistance [1, 2]. To overcome these major roadblocks to improve prognosis requires mechanism-based therapeutic strategies that maximize the antitumor effect of drugs while limiting their toxicities. These agents exert anticancer effects via multiple mechanisms, yet their most prominent mode of action involves the generation of DNA lesions followed by activation of the DNA damage response and induction of the BCL-2 family-dependent mitochondrial apoptosis [3, 4]. However, the signaling pathways that connect to BCL-2-family-regulated cell death are not entirely clear.

The BCL-2 family consists of three main groups of proteins: multi-domain anti-apoptotic (e.g., BCL-2, MCL-1, BCL-X_L_), multi-domain pro-apoptotic (e.g., BAX, BAK), and BH3-only pro-apoptotic (e.g., BAD, BID, BIM, Noxa). BH3-only proteins cause the release of cytochrome c from the mitochondria by activating BAX and/or BAK, while the anti-apoptotic BCL-2 family of proteins prevents this process [5, 6]. It has been shown that Noxa can bind and trigger proteasome-mediated MCL-1 degradation in response to UV irradiation or anticancer agents [7, 8]. Noxa is also transcriptionally induced by cisplatin, which is important for cell death in response to platinum-based drugs [9, 10]. However, the precise mechanisms of Noxa-induced cell death through MCL-1 degradation are obscure. Here we show that cisplatin-induced MCL-1 phosphorylation precedes its degradation. We have identified novel sites on MCL-1 that are specifically phosphorylated in response to Noxa expression. For regulation of phosphorylation, MCL-1 and CDK2 form a stable complex and further binding of Noxa to this complex enhances the phosphorylation. When the CDK2-mediated phosphorylation sites of MCL-1 were substituted with alanine, the mutated MCL-1 became more stable than wild-type and inhibited apoptosis induced by Noxa overexpression or cisplatin treatment. These findings identify a novel regulatory mechanism mediated by the Noxa/MCL-1/CDK2 complex, which could be a potential therapeutic target to overcome cellular resistance to DNA damaging agents.

## RESULTS

### DNA damaging agent-induced apoptosis is Noxa-dependent and phosphorylation of MCL-1 triggers MCL-1 reduction and apoptosis

**Figure 1:**
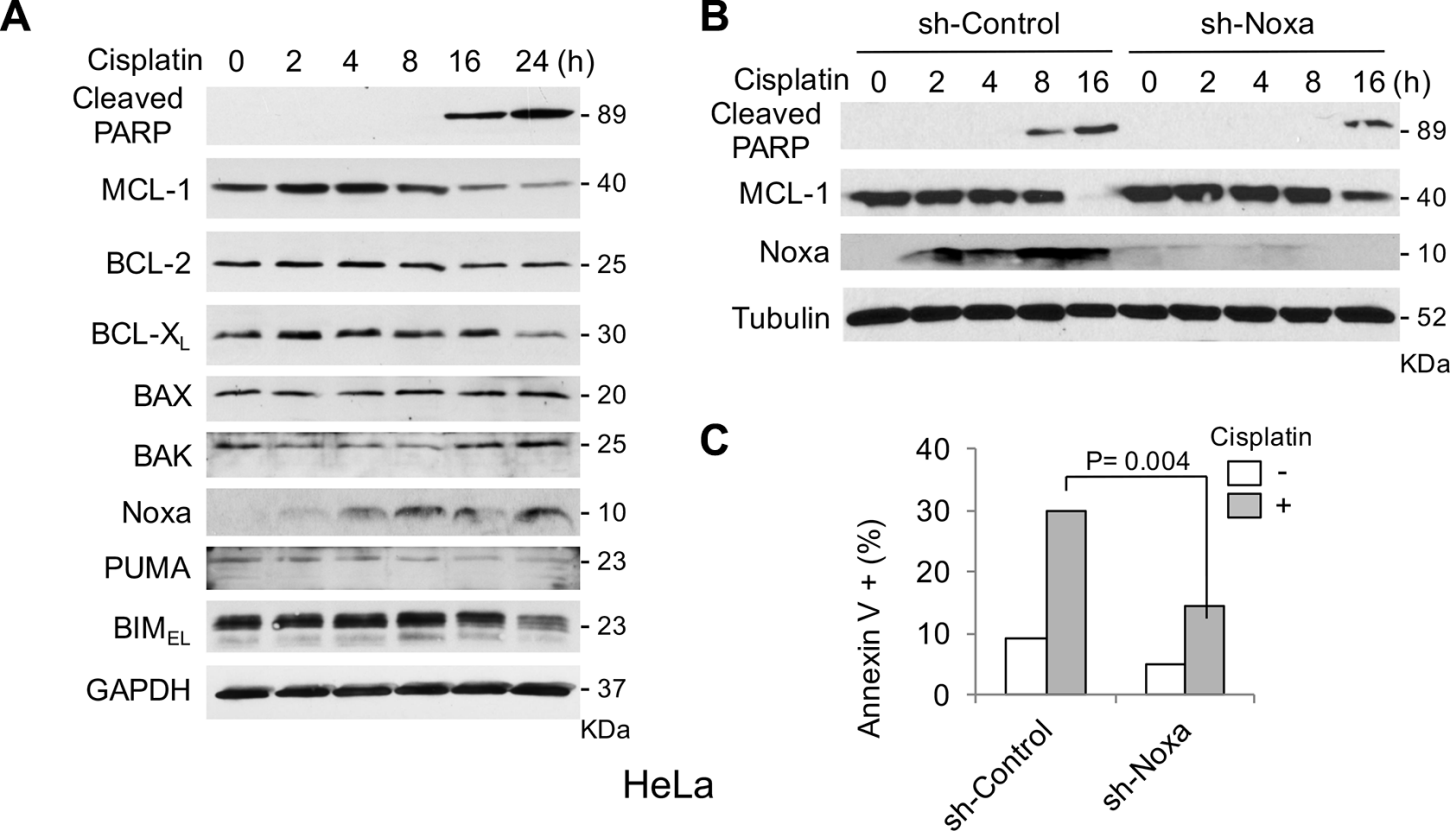
Noxa is required for cisplatin-induced apoptosis (**A**) HeLa cells were treated with cisplatin (30 μM) for the indicated periods and equal amounts of total extracts were subjected to immunoblot analysis using the indicated antibodies. (**B**) HeLa cells were infected with lentivirus-encoding shRNA for non-targeting control or Noxa. Cells were then treated with cisplatin (30 μM) for the indicated periods and equal amounts of total extracts were subjected to immunoblot analysis using the indicated antibodies. (**C**) HeLa cells in B were treated with cisplatin for 24 h and cell death was determined by Annexin V-PI staining followed by FACS analyses.

It has been demonstrated that treatment with DNA-damaging agents such as cisplatin induces Noxa expression and apoptosis in a variety of cell lines [9, 10]. Thus, to explore the significance of the Noxa-MCL-1 axis in cisplatin-induced cell death, we first determined the expression of the BCL-2 family proteins in cisplatin-treated HeLa cells (Figure 1A). PARP cleavage indicative of caspase activation and apoptosis was detected after 16 hours, while Noxa induction started 2 hours after the treatment and continued to increase to 24 hours. The level of MCL-1 started to decrease 8 hours prior to PARP cleavage and continued to decrease to 16–24 hours. In contrast, other BCL-2 family proteins were minimally affected by cisplatin treatment. The expression of both BIM and BCL-X_L_ was reduced at 24 hours when massive apoptosis was already observed. These results suggest that Noxa and MCL-1 may contribute to cisplatin-induced apoptosis.

We next examined whether Noxa is required for apoptosis induced by DNA damaging agents, such as cisplatin and etoposide. We stably expressed the shRNA for Noxa in HeLa cells and then treated with cisplatin over a 24 hour period. Down-regulation of Noxa resulted in a reduction of apoptosis induced by cisplatin or etoposide, as judged by PARP cleavage and annexin V staining (Figure 1B and 1C, Supplementary Figure S1A and S1B). Two other Noxa shRNAs also showed a reduction of cisplatin- or etoposide-induced apoptosis (Supplementary Figure S1C). Interestingly, the reduction of MCL-1 was also inhibited by down-regulation of Noxa. Cisplatin- or etoposide-induced Noxa up-regulation, MCL-1 reduction, and apoptosis were also observed not only in HeLa cervical cancer cells, but also in MDA-MB-468 breast cancer cells, HN30 head and neck squamous carcinoma cells, and mouse embryonic fibroblasts [11] (Supplementary Figure S1D, S1E, and S1F). Furthermore, down-regulation or deletion of Noxa attenuated MCL-1 reduction and apoptosis, suggesting that DNA damaging agents-mediated Noxa induction followed by MCL-1 reduction and apoptosis are common mechanisms.

**Figure 2:**
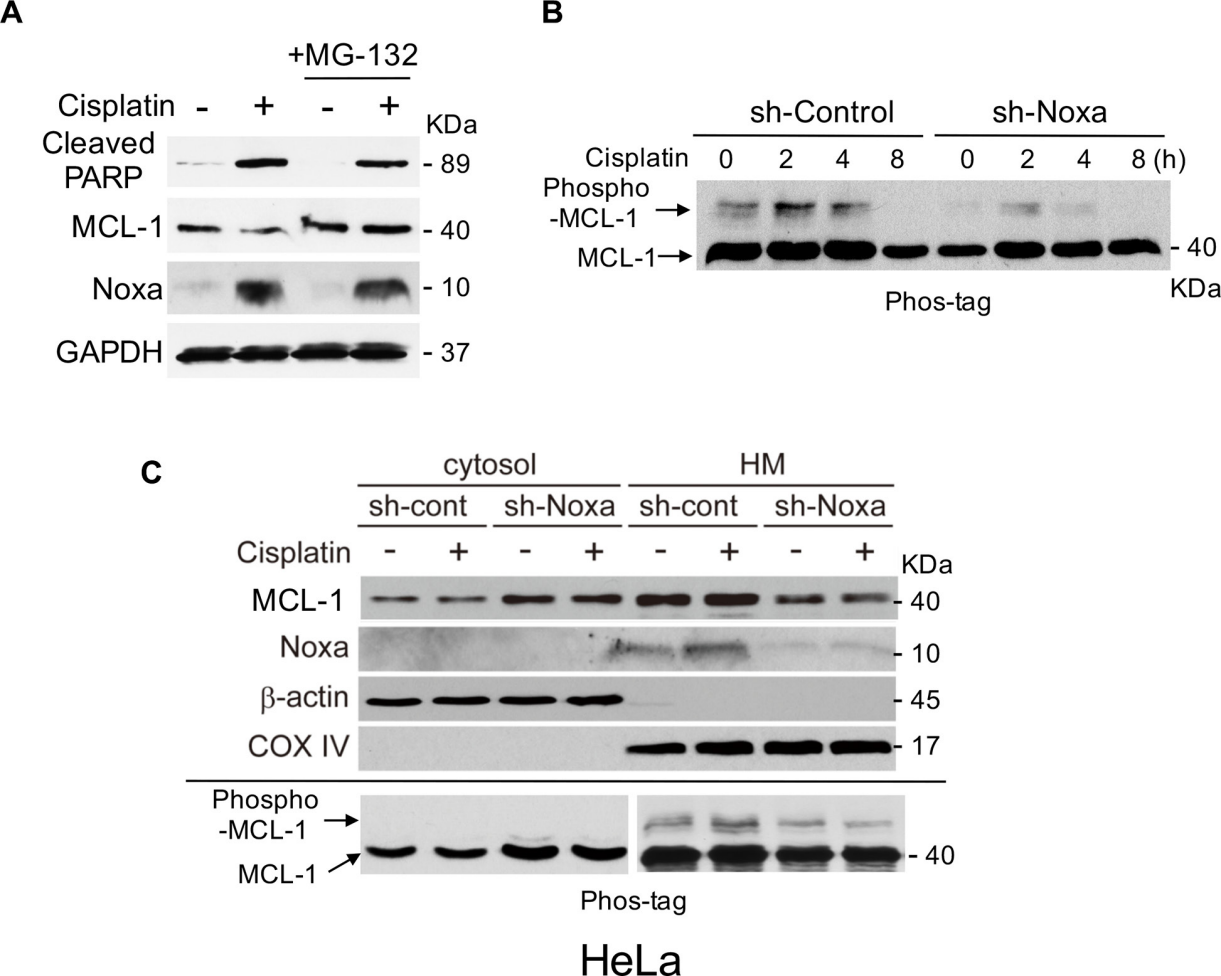
Noxa is required for cisplatin-induced MCL-1 phosphorylation and apoptosis (**A**) HeLa cells were treated with cisplatin (30 μM) for 18 h with or without MG-132 (10 μM) for 4 h before harvesting the cells. Equal amounts of total extracts were subjected to immunoblot analysis using the indicated antibodies. (**B**) HeLa cells were infected with lentivirus-encoding shRNA for non-targeting control or Noxa. Cells were then treated with cisplatin (30 μM) for the indicated periods and equal amounts of total extracts were applied in SDS-PAGE with 30 μM Phos-tag. (**C**) HeLa cells in B were treated with cisplatin for 4 h and were fractionated into cytosol and heavy membrane (HM). Each fraction was subjected to immunoblot analysis using the indicated antibodies.

The stability of MCL-1 is mainly regulated by phosphorylation followed by ubiquitination and proteasome-mediated degradation [12, 13]. We first determined whether cisplatin-mediated MCL-1 reduction is proteasome-dependent. The reduction of MCL-1 by cisplatin treatment was clearly blocked by a proteasome inhibitor, MG-132 (Figure 2A). Of note, the induction of Noxa and cleaved PARP was not affected significantly by MG-132, suggesting that MCL-1 reduction was caspase-independent. We next determined the phosphorylation status by using gels containing Phos-tag, which specifically binds to phosphate ions, resulting in decreased migration speed of phosphorylated proteins [14]. The amount of MCL-1 phosphorylation was increased at 2 hours after addition of cisplatin and then declined at 4–8 hours, which was decreased by down-regulation of Noxa (Figure 2B). Over the same period, the phosphorylation status of other anti-apoptotic BCL-2 family proteins, BCL-2 and BCL-X_L,_ were not altered (Supplementary Figure S2), suggesting that cisplatin-induced phosphorylation is MCL-1-specific.

We have previously shown that Noxa specifically binds to and recruits MCL-1 from the cytosol to the mitochondria. Translocation of MCL-1 initiates its phosphorylation and subsequent ubiquitination, which triggers proteasome-mediated degradation [15]. In the control cells, Noxa and MCL-1 were predominantly localized in the heavy membrane fraction and both were slightly increased with cisplatin treatment for 4 hours (Figure 2C). Down-regulation of Noxa by shRNA sharply decreased MCL-1 in the heavy membrane fraction and increased its presence in the cytosolic fraction, suggesting that MCL-1 is translocated through the interaction with Noxa. In the control cells, cisplatin-induced MCL-1 phosphorylation was observed in the heavy membrane fraction but not in the cytosolic fraction, the levels of which were reduced by down-regulation of Noxa (the lower panel in Figure 2C). These results support the concept that Noxa recruits MCL-1 to the mitochondria and initiates MCL-1 phosphorylation, which is augmented by cisplatin-mediated induction of Noxa.

### Noxa-induced MCL-1 phosphorylation is regulated by CDK2

**Figure 3:**
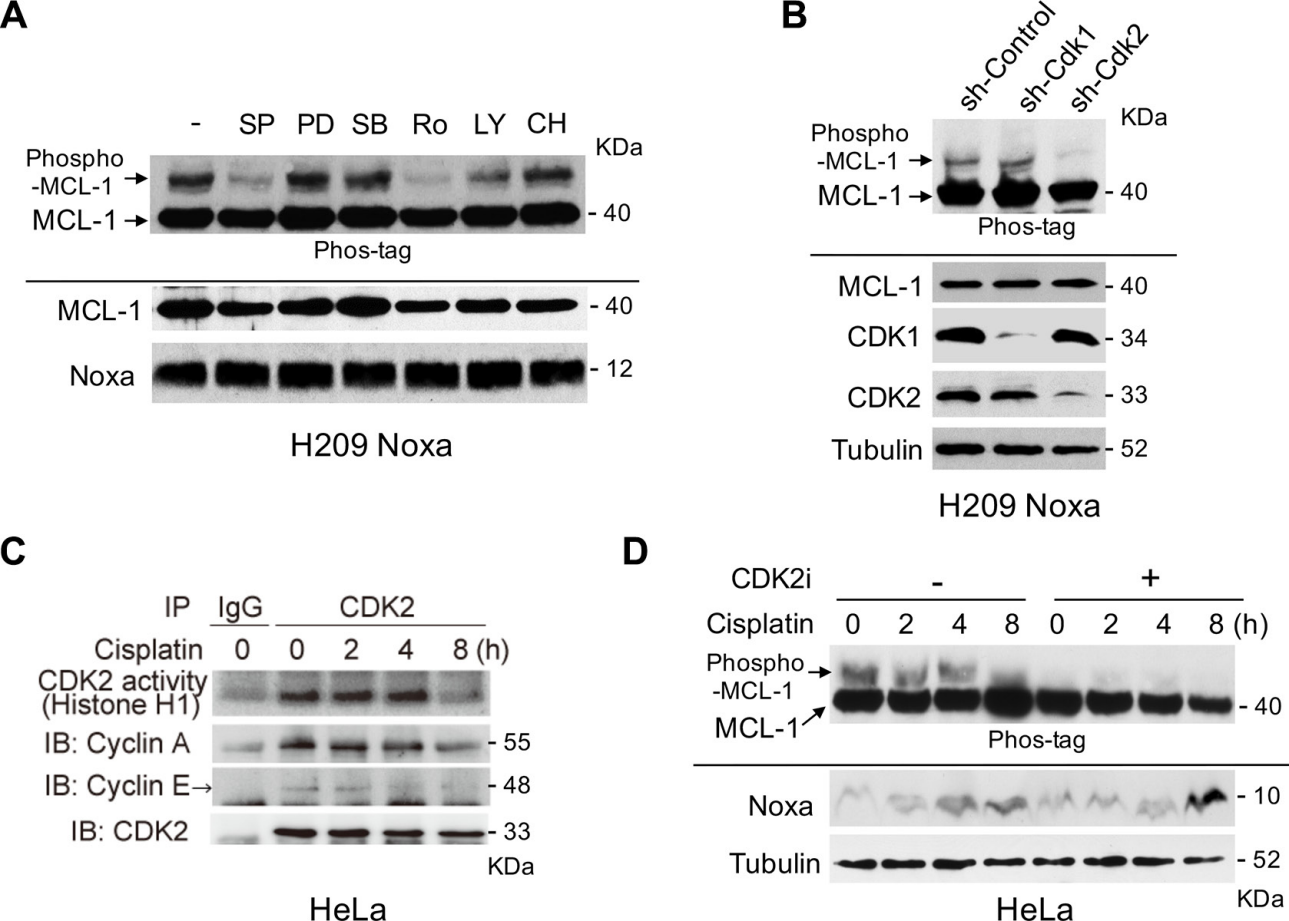
Noxa-induced MCL-1 phosphorylation is regulated by CDK2 (**A**) Flag-tagged human Noxa was expressed in Noxa-null H209 SCLC cells. Cells were treated with the indicated kinase inhibitors for 4 hours and phosphorylated MCL-1 was detected on SDS-PAGE with Phos-tag. SP: SP600125 (10 μM), PD: PD184352 (5 μM), SB: SB203580 (2 μM), Ro: Roscovitine (50 μM), LY: LY294002 (10 μM), CH: CHIR99021 (5 μM). (**B**) Lentivirus-mediated shRNA, as indicated, was introduced in the cells and phosphorylated MCL-1 was detected on SDS-PAGE with Phos-tag. (**C**) HeLa cells were treated with cisplatin for the indicated periods. Equal amounts of total extracts were immunoprecipitated with anti-CDK2 antibodies and CDK2 activities were measured by *in vitro* kinase assays with histone H1 as substrate. (**D**) HeLa cells were pretreated with Cdk2 inhibitor II for 30 min, and then treated with cisplatin for the indicated periods. Equal amounts of total extracts were subjected to immunoblot analysis using the indicated antibodies.

It has been shown that Noxa causes apoptosis by specifically interacting with MCL-1 to induce its proteasome-mediated degradation. This degradation is often regulated by the phosphorylation of MCL-1 followed by ubiquitination [12, 13]. Based on the above observation that cisplatin-induced MCL-1 phosphorylation and reduction were Noxa-dependent, we hypothesized that cisplatin-induced Noxa could directly regulate MCL-1 phosphorylation and stability. To identify the kinase(s) and the phosphorylation sites of MCL-1 induced by Noxa expression, we established stable expression of Flag-tagged human Noxa in H209 small cell lung cancer cells, in which the endogenous Noxa expression was undetectable [15]. We detected robust shifted bands of MCL-1 by using gels containing Phos-tag, indicating Noxa-induced MCL-1 phosphorylation (Figure 3A). We then treated the cells with various kinase inhibitors in an attempt to identify the kinase(s) involved in MCL-1 phosphorylation. SP600125 and Roscovitine strongly inhibited Noxa-induced MCL-1 phosphorylation (Figure 3A). However, a MEK inhibitor PD184352, a p38-MAPK inhibitor SB203580, a PI3K inhibitor LY294002, or a glycogen synthase kinase 3 (GSK3) inhibitor CHIR99021 did not affect MCL-1 phosphorylation to any discernible extent. Roscovitine has a broad specificity to inhibit CDKs and SP600125 was originally identified as a JNK inhibitor, but it non-specifically inhibits a variety of kinases including CDK2 [16]. Therefore, we speculated that CDK2 would be a likely kinase to mediate Noxa-induced MCL-1 phosphorylation. Using shRNA approaches, we confirmed the involvement of CDK2, but not CDK1, in Noxa-induced MCL-1 phosphorylation (Figure 3B).

We then determined cisplatin-induced CDK2 activity in HeLa cells. An *in vitro* kinase assay demonstrated that CDK2 was activated by cisplatin up to 4 hours after treatment (Figure 3C). The amounts of Cyclin A or Cyclin E co-precipitated with CDK2 and the CDK2 activity correlated well with the phosphorylation status of MCL-1 (sh-Control in Figure 2B and mock-CDK2 inhibitor in Figure 3D). In order to examine whether CDK2 contributes to the phosphorylation of MCL-1 induced by cisplatin treatment, HeLa cells were pretreated with a Cdk2 inhibitor II (CAS 222035-13-4) [17] and then treated with cisplatin over an 8 hour period, since the inhibitor itself was toxic to the cells beyond this time. The phosphorylation of MCL-1 induced by cisplatin was totally abolished by the CDK2 inhibitor, although Noxa was similarly induced with or without the inhibitor (Figure 3D). The data obtained from CDK2 shRNA experiments (Figure 3B) together with those generated using a CDK2 inhibitor (Figure 3D) strongly suggest that CDK2 contributes to Noxa-induced and cisplatin-induced MCL-1 phosphorylation.

### Ser64 and Thr70 on MCL-1 are phosphorylated when Noxa levels are increased

**Figure 4:**
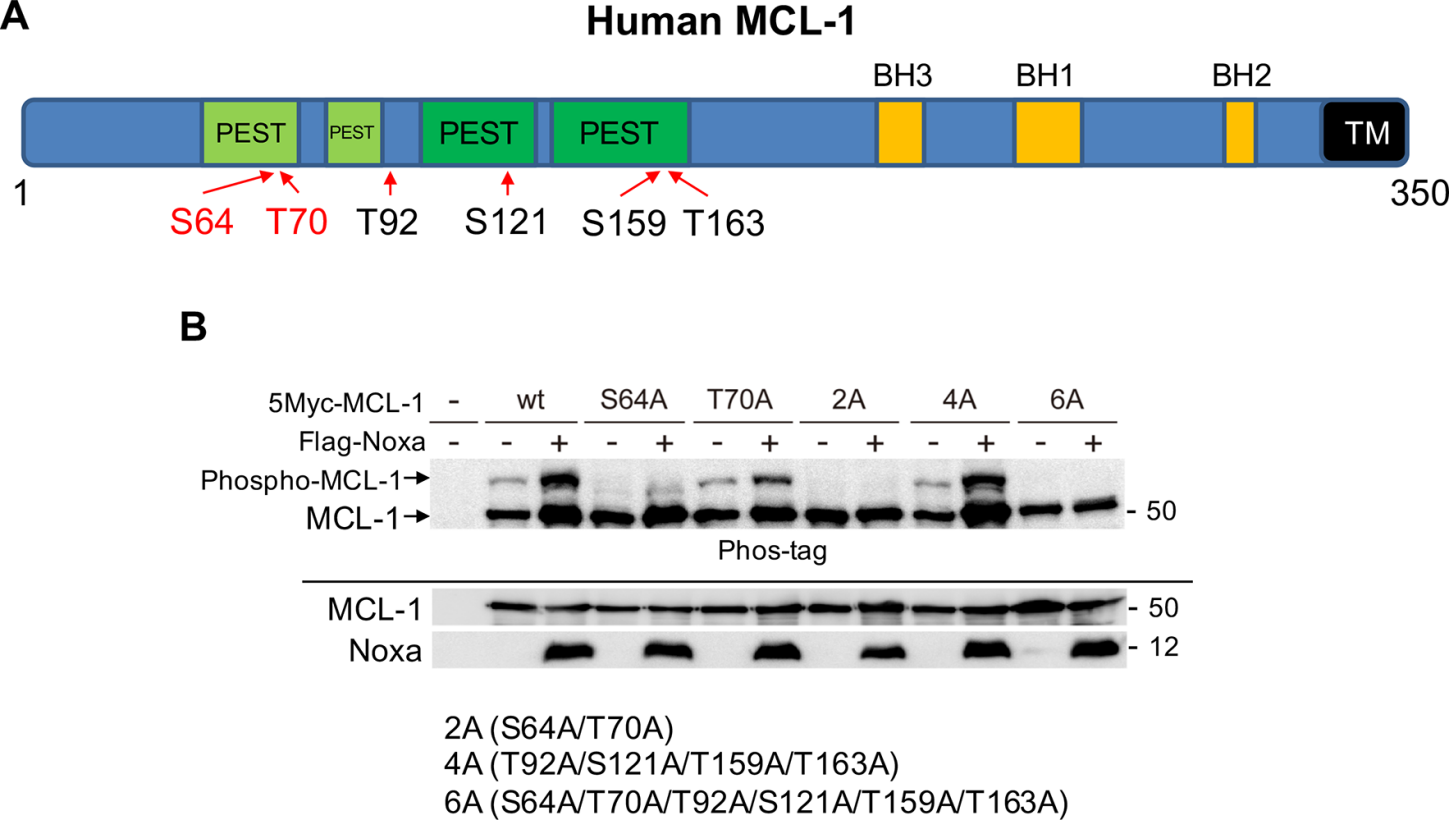
Ser64 and Thr70 on MCL-1 are phosphorylated by Noxa expression (**A**) Schematic representation of human MCL-1. Potential CDK2 phosphorylation sites are denoted. BH: BCL-2 homology domain; TM: transmembrane domain. (**B**) Myc-tagged human MCL-1 wild-type (wt) or the mutants in which Ser and/or Thr were substituted to Ala were co-transfected with human Noxa in 293T cells for 24 h. Equal amounts of total extracts were subjected to immunoblot analysis using the indicated antibodies.

We next determined the sites on MCL-1 that are phosphorylated by CDK2. There are 6 Ser-Pro or Thr-Pro sites in MCL-1 that are potentially phosphorylated by CDK2 (Figure 4A); thus, mutants were generated in which each Ser or Thr site was substituted to Ala and Noxa-induced phosphorylation was examined. We transiently co-transfected myc-tagged human MCL-1-wild-type (wt) or each mutant with human Noxa in 293T cells (Figure 4B). MCL-1-wt showed a Noxa-induced band shift on SDS-PAGE with phos-tag and the band migrated faster when Ser64 was substituted to Ala (S64A). The intensity of phosphorylated band of T70A (Thr70 was substituted to Ala) was reduced compared with that of MCL-1-wt. Other MCL-1 single site mutants showed Noxa-mediated band shifts similar to MCL-1-wt (Supplementary Figure S3). The MCL-1 mutant with Ser64 and Thr70 (MCL-1-2A) and MCL-1-6A, in which all Ser/Thr sites were substituted to Ala, lost Noxa-induced band shifts. However, a Noxa-mediated band-shift was detected similar to MCL-1-wt when Thr92, Ser121, Thr159, and Thr163 were mutated (MCL-1-4A) (Figure 4B). An *in vitro* kinase assay using active CDK2 and GST-MCL-1 as substrate confirmed that CDK2 could directly phosphorylate Ser64 and Thr70 on MCL-1 *in vitro* (Supplementary Figure S4A). In HeLa cells treated with cisplatin, Ser64 on MCL-1 was found to be phosphorylated using phospho-specific antibodies (Supplementary Figure S4B). These results indicate that these two amino acids are the major Noxa-induced MCL-1 phosphorylation sites.

### Noxa-mediated MCL-1 phosphorylation is required for MCL-1 ubiquitination and its stability

**Figure 5:**
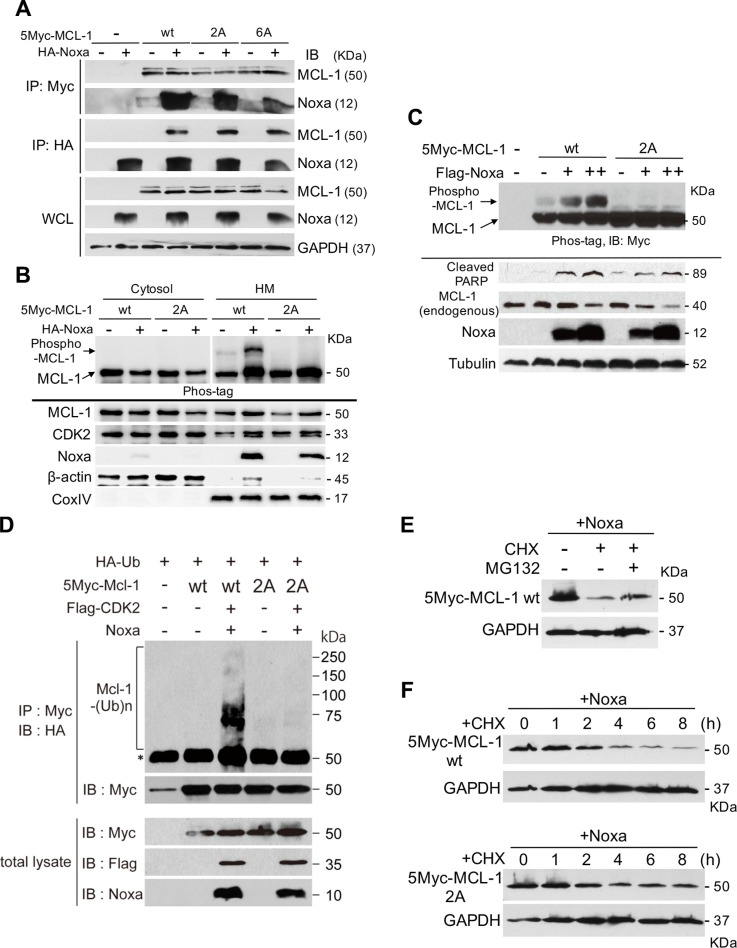
Ser64 and Thr70 contribute to the stability of MCL-1 (**A**) Myc-tagged human MCL-1 wt, the 2A mutant in which both Ser64 and Thr70 were substituted to Ala, or the 6A mutant in which all potential phosphorylation sites of Ser and Thr were substituted to Ala were co-transfected with Noxa in 293T cells for 24 h. The interaction between Noxa and MCL-1 was determined with co-immunoprecipitation followed by immunoblot analyses with the indicated antibodies. (**B**) The cells in A were fractionated into cytosol and heavy membrane (HM) fractions. Each fraction was subjected to immunoblot analysis using the indicated antibodies. (**C**) Myc-tagged human MCL-1 wt or the 2A mutant was stably expressed in HeLa cells. Flag-tagged human Noxa was transiently expressed for 24 h and total extracts were subjected to immunoblot analysis using the indicated antibodies. (**D**) HA-tagged ubiquitin, Myc-tagged MCL-1, Flag-tagged CDK2, and Noxa were transfected into HeLa cells for 24 h. Total extracts were immunoprecipitated with anti-Myc antibodies followed by immunoblotting with anti-HA antibodies to detect the ubiqutination of MCL-1. The asterisk (*) indicates the position of IgG heavy chain produced by immunoprecipitation. (**E**) Flag-tagged human Noxa was transiently expressed in the cells in C for 24 h. Then the cells were treated with cycloheximide (CHX, 10 μg/ml) for 6 h with or without MG-132 (10 μM) for 4 h before harvesting the cells. Equal amounts of total extracts were subjected to immunoblot analysis using the indicated antibodies. (**F**) Flag-tagged human Noxa was transiently expressed in the cells in C for 24 h. Then the cells were treated with cycloheximide (CHX, 10 μg/ml) for the indicated times. Equal amounts of total extracts were subjected to immunoblot analysis using the indicated antibodies.

We next assessed whether Noxa-mediated MCL-1 phosphorylation induces MCL-1 ubiqutination and determines the stability of this protein. When Noxa and MCL-1 wt or phosphorylation-site mutants were co-expressed in 293T cells, similar amounts of MCL-1 wt and mutants were bound to Noxa, as judged by co-immunoprecipitation (Figure 5A). We confirmed that Noxa-mediated MCL-1 phosphorylation was observed only in the mitochondria-enriched heavy membrane fraction when Ser64 and Thr70 of MCL-1 were intact (Figure 5B). Of note, CDK2 was localized in both cytosol and the heavy membrane fractions, supporting the possibility that CDK2 could phosphorylate MCL-1 at the mitochondria.

In order to examine the effect of MCL-1 phosphorylation in Noxa-induced apoptosis, we established HeLa cells that stably expressed myc-tagged MCL-1-wt or MCL-1-2A, and then increased amounts of Noxa were introduced into these cells. Increased MCL-1 phosphorylation was detected in accordance with the levels of Noxa expression only with MCL-1-wt, but not with MCL-1-2A (Figure 5C). The levels of MCL-1 2A were higher than those of MCL-1-wt, suggesting that the mutant is more stable than wt. Noxa-induced apoptosis, as judged by PARP cleavage, was increased in cells with MCL-1-wt, more so than in those with MCL-1-2A, suggesting that MCL-1-2A effectively inhibits Noxa-induced apoptosis. We further determined the Noxa-mediated MCL-1 ubiqutination status by transient co-expression with ubiquitin, Noxa, CDK2, and MCL-1. Ubiqutinated MCL-1 was only detected when Noxa and CDK2 were co-expressed with MCL-1-wt, but not with MCL-1-2A (Figure 5D). The ubiqutinated MCL-1 was clearly decreased with a protein synthesis inhibitor cycloheximide, and this was partially blocked by a proteasome inhibitor, MG-132 (Figure 5E), suggesting that the stability of MCL-1 is mainly regulated by proteasome-dependent mechanisms. We then compared MCL-1 stability between MCL-1-wt and −2A. MCL-1-2A showed a longer half-life than MCL-1-wt (Figure 5F). Taken together, the results clearly indicate that Ser64 and Thr70 in MCL-1 are required for Noxa-mediated MCL-1 phosphorylation by CDK2, ubiqitination, degradation, and consequently apoptosis.

### MCL-1-S64A/T70A inhibits cisplatin-induced apoptosis

**Figure 6:**
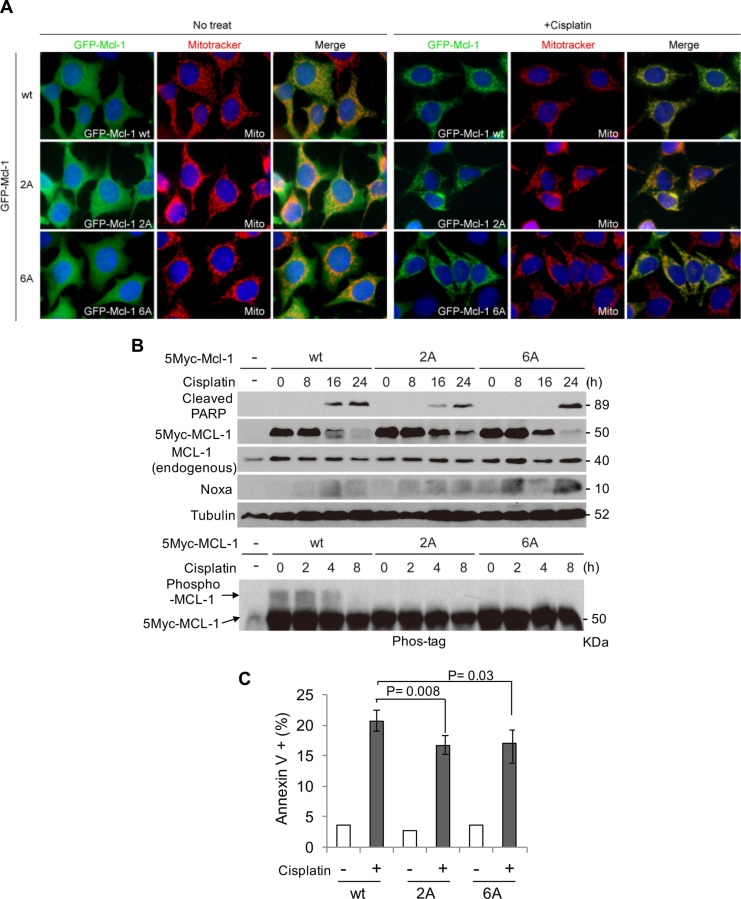
MCL-1 S64A/T70A inhibits cisplatin-induced apoptosis (**A**) GFP-tagged human MCL-1 wt, the 2A, or the 6A mutants were stably expressed in HeLa cells. Cells were treated with or without cisplatin for 10 h in the presence of MitoTracker Red and were visualized with a fluorescence microscope. Original magnification, ×400. (**B**) Myc-tagged human MCL-1 wt, the 2A, or the 6A mutants were stably expressed in HeLa cells. Cells were treated with cisplatin for the indicated periods and equal amounts of total extracts were subjected to immunoblot analysis using the indicated antibodies. (**C**) The cells in B were treated with cisplatin for 24 h and cell death was determined by Annexin V-PI staining followed by FACS analyses.

Noxa expression was capable of inducing apoptosis dependent on the MCL-1 phosphorylation status. We further explored whether the phosphorylation-site mutants of MCL-1 could inhibit cisplatin-induced apoptosis. HeLa cells that stably expressed GFP- or Myc-tagged MCL-1-wt, MCL-1-2A, or MCL-1-6A were treated with cisplatin. Co-localization of GFP-tagged MCL-1-wt and mutants with MitoTracker Red was significantly increased after cisplatin treatment (Figure 6A), indicating translocation of MCL-1 from cytosol to the mitochondria regardless of its phosphorylation status. However, MCL-1 phosphorylation induced by cisplatin was inhibited and as a consequence Myc-tagged MCL-1 degradation was delayed (particularly noticeable at 16 hours) in both MCL-1-2A and MCL-1-6A mutants (Figure 6B). Furthermore, cisplatin-induced apoptosis was also reduced in cells expressing MCL-1-2A or MCL-1-6A mutants (Figure 6C). Taken together, cisplatin-induced MCL-1 phosphorylation affects its stability and, as a consequence, apoptosis.

### CDK2 binds to and phosphorylates MCL-1, which is enhanced by Noxa expression

**Figure 7:**
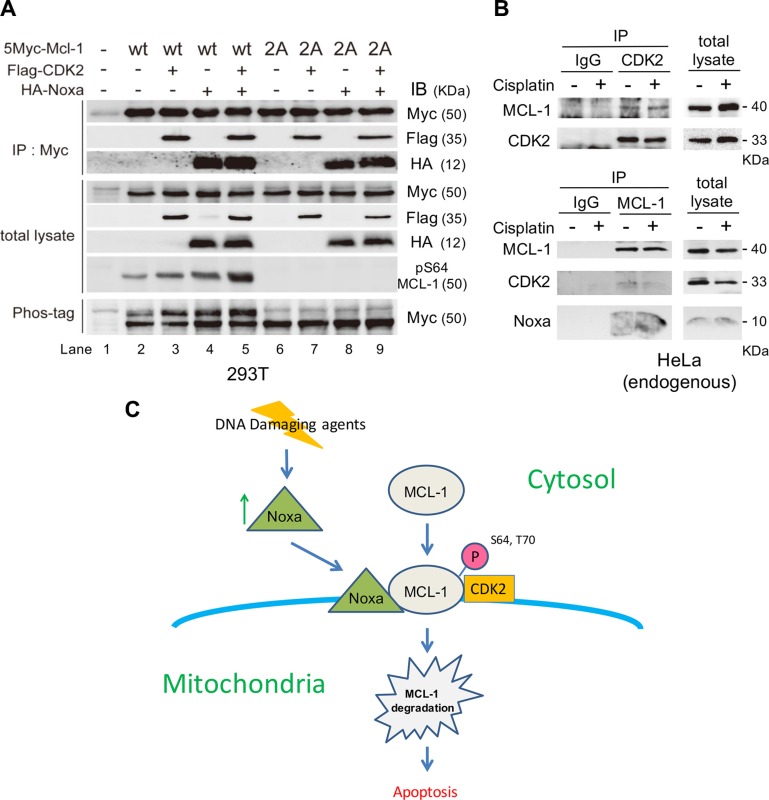
CDK2 binds to and phosphorylates MCL-1, which is enhanced by Noxa expression (**A**) HA-tagged Noxa, Myc-tagged MCL-1 (wt or 2A), and/or Flag-tagged CDK2 were transfected into 293T cells for 24 h. Total extracts were immunoprecipitated with anti-Myc antibodies followed by immunoblotting with the indicated antibodies to detect the molecular interactions. Phosphorylation of MCL-1 was detected with phospho-Ser64 specific antibodies or on SDS-PAGE with Phos-tag. (**B**) HeLa cells were treated with cisplatin for 4 h. Total extracts were immunoprecipitated with anti-CDK2 or anti-MCL-1 antibodies followed by immunoblotting with the indicated antibodies to detect the molecular interactions. (**C**) Regulation of apoptosis induced by DNA damaging agents through Noxa/MCL-1 CDK2 complex. Details are in the text.

In order to define the mechanisms through which CDK2-mediated MCL-1 phosphorylation is induced by Noxa, epitope-tagged Noxa, MCL-1 and CDK2 were co-expressed in 293T cells and Myc-tagged MCL-1 was immunoprecipitated. Using the cell lysates in which Myc-tagged MCL-1 and Flag-tagged CDK2 were co-expressed, we specifically detected transfected CDK2 (Figure 7A, lane 3). Similarly, co-expression of MCL-1 and Noxa showed strong interaction (Figure 7A, lane 4). When MCL-1, CDK2, and Noxa were co-expressed together, MCL-1 could co-precipitate both CDK2 and Noxa, but the amount of precipitated CDK2 or Noxa was not changed compared to that in MCL-1/CDK2 or MCL-1/Noxa co-expression (Figure 7A, lanes 3–5), suggesting that MCL-1/CDK2 interaction occurs through a domain distinct from that required for MCL-1/Noxa interaction, which is mediated through the Noxa BH3 domain. When MCL-1 2A (Ser64A/Thr70A) was introduced, the interaction with CDK2 and Noxa was similar to that when MCL-1-wt was expressed, suggesting that the phosphorylation sites are not involved in the interaction. The introduction of MCL-1-wt in 293T cells led to a band shift on SDS-PAGE with phos-tag, indicating MCL-1 phosphorylation by an endogenous kinase(s). The amount of phosphorylation was increased by exogenous CDK2 expression and further increased by co-expression of Noxa (Figure 7A, lanes 2–5), suggesting that CDK2 binds to and phosphorylates MCL-1, which is enhanced by Noxa expression. The amount of Ser64 phosphorylation was confirmed by MCL-1 phospho-Ser64 specific antibodies. Of note, the MCL-1 2A mutant did not show any phosphorylation of MCL-1, confirming specificity. When Noxa 3E, a BH3-domain mutant that is unable to bind to MCL-1, was co-expressed, the amount of MCL-1 phosphorylation was not increased as observed with Noxa-wt (Supplementary Figure S5), indicating that the interaction between Noxa and MCL-1 is required for maximal MCL-1 phosphorylation. Finally, we confirmed the interaction between MCL-1, CDK2, and Noxa at the endogenous level in HeLa cells (Figure 7B). These results suggest that Noxa, MCL-1 and CDK2 form a complex to enhance the phosphorylation of MCL-1 followed by MCL-1 degradation, which is a mechanism of Noxa-induced apoptosis.

## DISCUSSION

It has been well established that Noxa, a BH3-only protein, can bind to MCL-1 with high affinity and trigger proteasome-mediated MCL-1 degradation [18]. However, the precise mechanism of this regulation is unclear. Here, we report that Noxa is an important mediator of apoptosis induced by DNA damaging agents, cisplatin and etoposide in HeLa (cervical), HN30 (head and neck), and MDA-MB-468 (breast) tumor cells (Figure 1 and Supplementary Figure S1). Mechanistically, Noxa is upregulated by cisplatin treatment, followed by CDK2-mediated MCL-1 phosphorylation, ubiqutination, and degradation (Figure 7C). As a consequence, apoptosis is induced efficiently. We identified novel Noxa-mediated phosphorylation sites on MCL-1, namely Ser64 and Thr70. Several phosphorylation sites on MCL-1 have been identified, all of which contribute to the stability of MCL-1 [19]. However, the kinases and the consequence of phosphorylation are context-dependent. It has been shown that CDK2 can phosphorylate Ser64 on MCL-1 *in vitro*, which does not affect its stability, but enhances the MCL-1 pro-survival function [20]. Another recent manuscript has demonstrated that Cyclin E/CDK2-dependent phosphorylation of MCL-1 at Ser64, Thr92, and Thr163 results in increased MCL-1 stability [21]. Others have shown that CDK1 phosphorylates MCL-1 at Thr92, resulting in MCL-1 degradation and that CDK1 is required for Ser64 phosphorylation in mitosis [22]. We show here that MCL-1 phosphorylation was increased by cisplatin treatment in concordance with the activity of CDK2 (Figures 2B and 3C). We confirmed that CDK2 could directly phosphorylate Ser64 and Thr70 on MCL-1 *in vitro* and Ser64 was phosphorylated following cisplatin treatment (Supplementary Figure S4). The phosphorylation of Ser64 and Thr70 sites was required for ubiquitination of MCL-1 (Figure 5D). When these sites were substituted with Ala to become non-phosphorylatable, the mutated MCL-1 was more stable than MCL-1-wt (Figure 5F). These results strongly suggest that phosphorylation of Ser64 and Thr70 destabilizes MCL-1 through a proteasome-dependent pathway.

CDK2 and MCL-1 stably bound to each other and Noxa expression facilitated MCL-1 phosphorylation without affecting the CDK2/MCL-1 interaction (Figure 7A). However, the phosphorylation status of MCL-1 at Ser64 and Thr70 did not affect the binding capacity between MCL-1 and Noxa (Figures 5A and 7A). These results suggest that the protein-protein interaction sites for CDK2/MCL-1 and Noxa/MCL-1 are distinct and the binding of Noxa may induce conformational change of the CDK2/MCL-1 complex, making the CDK2-mediated Ser64/Thr70 phosphorylation sites on MCL-1 more accessible. It has been demonstrated that the BH3-binding pocket on MCL-1, to which Noxa binds, is localized at amino acids 172–320 [23]. Thus, we speculate that CDK2 may bind to the N-terminal region of MCL-1 (amino acids 1–171) that includes the CDK2 phosphorylation sites at Ser64 and Thr70.

The role of CDK2 in cell death is controversial; some reports indicate that the activation of CDK2 is pro-apoptotic, but the others indicate that it may be pro-survival [24, 25]. Several studies have demonstrated that inhibition of CDK2 leads to cisplatin-resistance [26, 27]. Consistent with these observations, our results clearly indicate that CDK2 activated by cisplatin treatment has a pro-apoptotic function that is mediated through MCL-1 phosphorylation and degradation. Although we did not detect phosphorylation of BCL-X_L_ after cisplatin treatment in HeLa cells (Supplementary Figure S2), it has recently been shown that CDK2 phosphorylates BCL-X_L_ to convert its activity from anti-apoptotic to pro-apoptotic in mouse kidney proximal tubule epithelial cells [28]. These results suggest that CDK2 plays a pro-apoptotic role in cisplatin treatment, although the substrates of phosphorylation may be cell-type dependent. In the normal cell cycle, CDK2 forms a complex with Cyclin A or Cyclin E predominantly in the S phase or the G1 phase, respectively. Thus, the status of MCL-1 phosphorylation and stability could also be modulated during the normal cell cycle, which may contribute to the regulation of the balance between cell cycle progression and apoptosis.

In conclusion, we have defined a unique molecular mechanism for the regulation of MCL-1 and initiation of apoptosis induced by DNA damaging agents. We have identified CDK2 as a crucial kinase that binds and directs MCL-1 phosphorylation and degradation following cisplatin treatment, and also determined novel phosphorylation sites, Ser64 and Thr70 through which these effects are mediated. Although DNA damaging agent-mediated Noxa induction followed by MCL-1 reduction and apoptosis are common mechanisms (Figure 1 and Supplementary Figure S1), it remains undetermined that CDK2 contributes to this process in general. It is of interest in the future to analyze whether CDK2 expression/activity correlates with cisplatin-resistance in patient samples.

## MATERIALS AND METHODS

### Cell lines and cell culture

HeLa, 293T, MDA-MB-468, and H209 cells were purchased from the American Type Culture Collection (Manassas, VA, USA). Mouse embryonic fibroblasts (MEFs) were prepared as previously described [11]. HN30 cells have been described in detail elsewhere [29]. H209 cells were cultured in RPMI1640 (Life Technologies, Grand Island, NY) supplemented with 10% heat-inactivated fetal bovine serum (FBS) and 100 μg/ml penicillin G/streptomycin at 37°C in a humidified, 5% CO_2_ incubator. Other cell lines were cultured in DMEM (Life Technologies) supplemented with 10% FBS and 100 μg/ml penicillin G/streptomycin.

### Plasmid construction and virus infection

The lentiviral short-hairpin RNA (shRNA)-expressing constructs were purchased from Open Biosystems (Huntsville, AL, USA) or Sigma-Aldrich (St. Louis, MO, USA). The target sequences for Noxa shRNA are the following; Noxa #1: 5′-CTACTCAACTCAGGAGAT-3′ (Supplementary Figure S1C), #2: 5′-GGAAACGGAAGATGGAATA-3′ (Supplementary Figure S1C), 5′-CTTCCGGCAGAAACT TCTGAA-3′ (other Figures). Human Noxa and Mcl-1 expression vectors were generated in our previous study [15]. The N-terminal GFP-tagged human MCL-1 (GFP-MCL-1) was constructed by cloning *Mcl-1* cDNA into pBabe-puro encoding a GFP epitope tag. *Mcl-1* mutant cDNAs were constructed with QuickChange Lightning Site-Directed Mutagenesis kit (Agilent Technologies, Santa Clara, CA) according to the manufacturer's protocol. The constructs were transfected into 293T packaging cells along with the packaging plasmids and the lentivirus- or retrovirus-containing supernatants were used to transduce the cells.

Mouse *Cdk2* cDNA was cloned from MEFs by RT-PCR. The N-terminal Flag-tagged mouse CDK2 (Flag-CDK2) was constructed by cloning the above *Cdk2* cDNA into pcDNA3-Flag (Life Technologies). The expression vector pcDNA3-HA-Ubiquitin was kindly provided by Fuminori Tokunaga (Gunma University, Maebashi, Gunma, Japan).

### Chemicals and antibodies

Reagents were purchased as follows: cisplatin from Santa Cruz Biotechnology (Santa Cruz, CA, USA); etoposide and SP600125 from LC Laboratories (Woburn, MA, USA); SB203580, Roscovitine, LY294002, and CHIR99021 from Sigma-Aldrich; Cycloheximide and MG-132 from Millipore (Billerica, MA, USA); Phos-tag acrylamide from Wako Chemicals USA (Richmond, VA). PD184352 was kindly provided by Steven Grant (Virginia Commonwealth University), which was chemically synthesized in house based on the published structure of the drug. Cisplatin was dissolved in PBS [30] and other reagents were dissolved in dimethyl sulfoxide. The following antibodies were used: Cleaved PARP (D64E10), Cleaved Caspase-3 (5A1E), BCL-X_L_, BIM, phospho-S64 MCL-1, COX IV (3E11), β-Actin (13E5) and GAPDH from Cell Signaling Technology (Danvers, MA, USA); Noxa (114C307.1) from Thermo Fisher Scientific (Waltham, MA, USA); MCL-1 from Enzo Life Sciences (Farmingdale, NY, USA) and Rockland Immunochemicals (Gilbertville, PA, USA); BAK (06-536) from Millipore; BAX (N-20), alpha-Tubulin (sc-8035), Myc (9E10) and CDK1 (sc-54) from Santa Cruz Biotechnology; CDK2 from BD Biosciences; HA (3F10) from Roche (Indianapolis, IN, USA). [γ-^32^P]ATP was purchased from PerkinElmer (Kanagawa, Japan). Histone H1, recombinant CDK2/Cyclin A, and CDK2/Cyclin E were purchased from SignalChem (Richmond, BC, Canada).

### Immunoprecipitation and immunoblot analyses

Whole cell lysates were prepared with CHAPS lysis buffer [20 mM Tris (pH 7.4), 137 mM NaCl, 1mM dithiothreitol (DTT), 1% CHAPS (3-[(3-cholamidopropyl) dimethylammonio]-1-propanesulfonate), a protease inhibitor cocktail, and phosphatase inhibitor cocktails (Sigma)]. For immunoprecipitation, equal amounts of protein were pre-cleared with protein A/G beads (Pierce, Rockford, IL, USA) and incubated with the appropriate antibodies on ice for 2 h. Then the antibody complexes were captured with protein A/G beads at 4^°^C for 1 h. After washing three times with the same lysis buffer, the beads were re-suspended in the sample buffer and separated by SDS-PAGE. For immunoblotting analyses, equal amounts of proteins were loaded on a SDS acrylamide gel, transferred to a nitrocellulose membrane, and analyzed by immunoblotting. Phosphorylated MCL-1 was detected on 8% SDS-PAGE with 30 μM Phos-tag acrylamide according to the manufacturer's protocol.

### Subcellular fractionation

Subcellular fractionation was performed using the Qproteome Cell Compartment kit according to the manufacture's protocol (Qiagen, Valencia, CA, USA). The pellet was re-suspended in CHAPS lysis buffer for a heavy-membrane (HM) fraction and the supernatant was used as a cytosolic fraction.

### Cell viability assay

Cell death was quantified by Annexin-V-FITC (BD Biosciences, San Jose, CA, USA)-propidium iodide (Sigma) staining according to the manufacturer's protocol, followed by flow cytometric analysis using FACScan (BD Biosciences).

### Immunofluorescence analysis

Cells were plated on sterile glass-bottom 96 well plates (Iwaki Glass, Tokyo, Japan) for 24 h and then treated with cisplatin for 10 h in the presence of 0.1 μM Mito Tracker Red CMXRos (Molecular Probes) and 10 μM Q-VD-OPH (a caspase inhibitor). Cells were washed with PBS, fixed with 4% paraformaldehyde in PBS for 30 min and methanol for 30 min, and washed three times with PBS. Hoechst 33342 (Invitrogen, Carlsbad, CA, USA) was added to a final concentration of 0.1 μg/ml for 10 min at room temperature. Cells were then washed with PBS and were observed using the Olympus IX71 fluorescence microscope (Olympus Corporation, Tokyo, Japan). Images were captured using the same exposure setting in each experiment.

### Quantitative RT-PCR

Quantitative real-time PCR analysis was performed as previously described [15]. The primer and probe sets (β-actin, Mm00607939_s1; Noxa, Mm00451763_ml) were purchased from Applied Biosystems (Carlsbad, CA, USA). Data were analyzed as mRNA expression levels relative to β-actin according to the manufacturer's protocol.

### GST-MCL-1 proteins and *in vitro* kinase assays

Cell lysates were immunoprecipitated with CDK2 antibodies using protein G sepharose beads. The beads were incubated in 50 μl of the kinase assay buffer [50 mM Tris (pH 7.5), 10 mM MgCl_2_, 1 mM DTT, 1 mM EGTA, 50 mM β-glycerophosphate, 25 mM NaF, 0.1 mM Na_3_VO_4_] containing 1 μg of Histone H1 and 5 μCi [γ-^32^P]ATP for 30 min at 30°C and then subjected to 10% SDS-PAGE. The gel was exposed to autoradiography. For preparation of GST-MCL-1 proteins, the recombinant plasmids pGEX6P-1 MCL-1-wt or MCL-1-2A were transformed into BL21 CodonPlus (DE3)-RIL (Agilent Technologies,). GST-fusion MCL-1 proteins were bound to Glutathione Sepharose 4B beads (GE Healthcare) using Bacterial Protein Extraction Reagent Buffer (Thermo Fisher Scientific). The beads were then incubated with the kinase assay buffer in the presence of 5 μCi [γ-^32^P]ATP and reconstituted 0.1 μg of CDK2/CycA or CDK2/CycE for 30 min at 30°C and then subjected to SDS-PAGE followed by Coomassie Brilliant Blue (CBB) staining (Nacalai, Tokyo, Japan). The gel was exposed to autoradiography after CBB staining.

### Statistical analysis

Values represent the means ± S.D. for three separate experiments. The significance of differences between the experimental variables was determined using the Student's *t*-test. Values were considered statistically significant at *P* < 0.05.

## SUPPLEMENTARY MATERIALS FIGURES



## References

[1] Galluzzi L, Senovilla L, Vitale I, Michels J, Martins I, Kepp O, Castedo M, Kroemer G (2012). Molecular mechanisms of cisplatin resistance. Oncogene.

[2] Galluzzi L, Vitale I, Michels J, Brenner C, Szabadkai G, Harel-Bellan A, Castedo M, Kroemer G (2014). Systems biology of cisplatin resistance: past, present and future. Cell Death Dis.

[3] Siddik ZH (2003). Cisplatin: mode of cytotoxic action and molecular basis of resistance. Oncogene.

[4] Brozovic A, Ambriovic-Ristov A, Osmak M (2010). The relationship between cisplatin-induced reactive oxygen species, glutathione, and BCL-2 and resistance to cisplatin. Crit Rev Toxicol.

[5] Youle RJ, Strasser A (2008). The BCL-2 protein family: opposing activities that mediate cell death. Nat Rev Mol Cell Biol.

[6] Chipuk JE, Moldoveanu T, Llambi F, Parsons MJ, Green DR (2010). The BCL-2 family reunion. Mol Cell.

[7] Nijhawan D, Fang M, Traer E, Zhong Q, Gao W, Du F, Wang X (2003). Elimination of Mcl-1 is required for the initiation of apoptosis following ultraviolet irradiation. Genes Dev.

[8] Inoue S, Riley J, Gant TW, Dyer MJ, Cohen GM (2007). Apoptosis induced by histone deacetylase inhibitors in leukemic cells is mediated by Bim and Noxa. Leukemia.

[9] Sheridan C, Brumatti G, Elgendy M, Brunet M, Martin SJ (2010). An ERK-dependent pathway to Noxa expression regulates apoptosis by platinum-based chemotherapeutic drugs. Oncogene.

[10] Zhu Y, Regunath K, Jacq X, Prives C (2013). Cisplatin causes cell death via TAB1 regulation of p53/MDM2/MDMX circuitry. Genes Dev.

[11] Nakajima W, Tanaka N (2011). Noxa induces apoptosis in oncogene-expressing cells through catch-and-release mechanism operating between Puma and Mcl-1. Biochem Biophys Res Commun.

[12] Inuzuka H, Fukushima H, Shaik S, Liu P, Lau AW, Wei W (2011). Mcl-1 ubiquitination and destruction. Oncotarget.

[13] Mojsa B, Lassot I, Desagher S (2014). Mcl-1 ubiquitination: unique regulation of an essential survival protein. Cells.

[14] Kinoshita E, Kinoshita-Kikuta E, Takiyama K, Koike T (2006). Phosphate-binding tag, a new tool to visualize phosphorylated proteins. Mol Cell Proteomics.

[15] Nakajima W, Hicks MA, Tanaka N, Krystal GW, Harada H (2014). Noxa determines localization and stability of MCL-1 and consequently ABT-737 sensitivity in small cell lung cancer. Cell Death Dis.

[16] Bain J, McLauchlan H, Elliott M, Cohen P (2003). The specificities of protein kinase inhibitors: an update. Biochem J.

[17] Davis ST, Benson BG, Bramson HN, Chapman DE, Dickerson SH, Dold KM, Eberwein DJ, Edelstein M, Frye SV, Gampe Jr RT, Griffin RJ, Harris PA, Hassell AM (2001). Prevention of chemotherapy-induced alopecia in rats by CDK inhibitors. Science.

[18] Czabotar PE, Lee EF, van Delft MF, Day CL, Smith BJ, Huang DC, Fairlie WD, Hinds MG, Colman PM (2007). Structural insights into the degradation of Mcl-1 induced by BH3 domains. Proc Natl Acad Sci U S A.

[19] Thomas LW, Lam C, Edwards SW (2010). Mcl-1; the molecular regulation of protein function. FEBS Lett.

[20] Kobayashi S, Lee SH, Meng XW, Mott JL, Bronk SF, Werneburg NW, Craig RW, Kaufmann SH, Gores GJ (2007). Serine 64 phosphorylation enhances the antiapoptotic function of Mcl-1. J Biol Chem.

[21] Choudhary GS, Tat TT, Misra S, Hill BT, Smith MR, Almasan A, Mazumder S (2015). Cyclin E/Cdk2-dependent phosphorylation of Mcl-1 determines its stability and cellular sensitivity to BH3 mimetics. Oncotarget.

[22] Harley ME, Allan LA, Sanderson HS, Clarke PR (2010). Phosphorylation of Mcl-1 by CDK1-cyclin B1 initiates its Cdc20-dependent destruction during mitotic arrest. EMBO J.

[23] Stewart ML, Fire E, Keating AE, Walensky LD (2010). The MCL-1 BH3 helix is an exclusive MCL-1 inhibitor and apoptosis sensitizer. Nat Chem Biol.

[24] Huang H, Regan KM, Lou Z, Chen J, Tindall DJ (2006). CDK2-dependent phosphorylation of FOXO1 as an apoptotic response to DNA damage. Science.

[25] Choi JS, Shin S, Jin YH, Yim H, Koo KT, Chun KH, Oh YT, Lee WH, Lee SK (2007). Cyclin-dependent protein kinase 2 activity is required for mitochondrial translocation of Bax and disruption of mitochondrial transmembrane potential during etoposide-induced apoptosis. Apoptosis.

[26] Price PM, Yu F, Kaldis P, Aleem E, Nowak G, Safirstein RL, Megyesi J (2006). Dependence of cisplatin-induced cell death *in vitro* and *in vivo* on cyclin-dependent kinase 2. J Am Soc Nephrol.

[27] Koster R, di Pietro A, Timmer-Bosscha H, Gibcus JH, van den Berg A, Suurmeijer AJ, Bischoff R, Gietema JA, de Jong S (2010). Cytoplasmic p21 expression levels determine cisplatin resistance in human testicular cancer. J Clin Invest.

[28] Megyesi J, Tarcsafalvi A, Seng NS, Hodeify R, Price PM (2016). Cdk2 phosphorylation of Bcl-xL after stress converts it to a pro-apoptotic protein mimicking Bax/Bak. Cell Death Discovery.

[29] Cardinali M, Pietraszkiewicz H, Ensley JF, Robbins KC (1995). Tyrosine phosphorylation as a marker for aberrantly regulated growth-promoting pathways in cell lines derived from head and neck malignancies. Int J Cancer.

[30] Hall MD, Telma KA, Chang KE, Lee TD, Madigan JP, Lloyd JR, Goldlust IS, Hoeschele JD, Gottesman MM (2014). Say no to DMSO: dimethylsulfoxide inactivates cisplatin, carboplatin, and other platinum complexes. Cancer Res.

