# Supplementary material for: DNA damaging agent-induced apoptosis is regulated by MCL-1 phosphorylation and degradation mediated by the Noxa/MCL-1/CDK2 complex
Source: Oncotarget. 2016 May 7;7(24):36353–65. doi: 10.18632/oncotarget.9217 (PMC5095005; doi:10.18632/oncotarget.9217)
Supplement: Supplementary file 1 [file oncotarget-07-36353-s001.pdf]

# DNA damaging agent-induced apoptosis is regulated by MCL-1 phosphorylation and degradation mediated by the Noxa/MCL-1/CDK2 complex

## Supplementary Materials

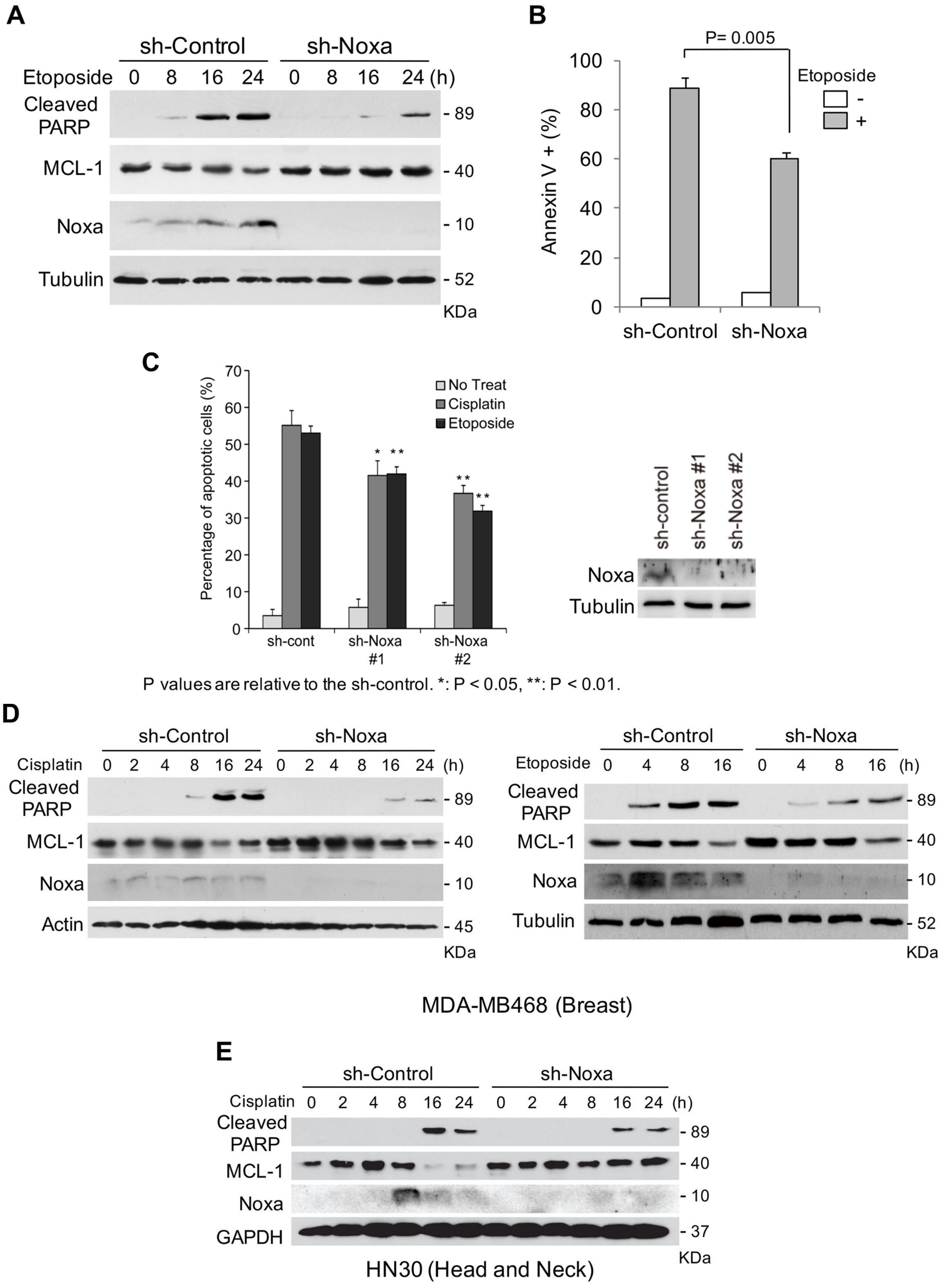

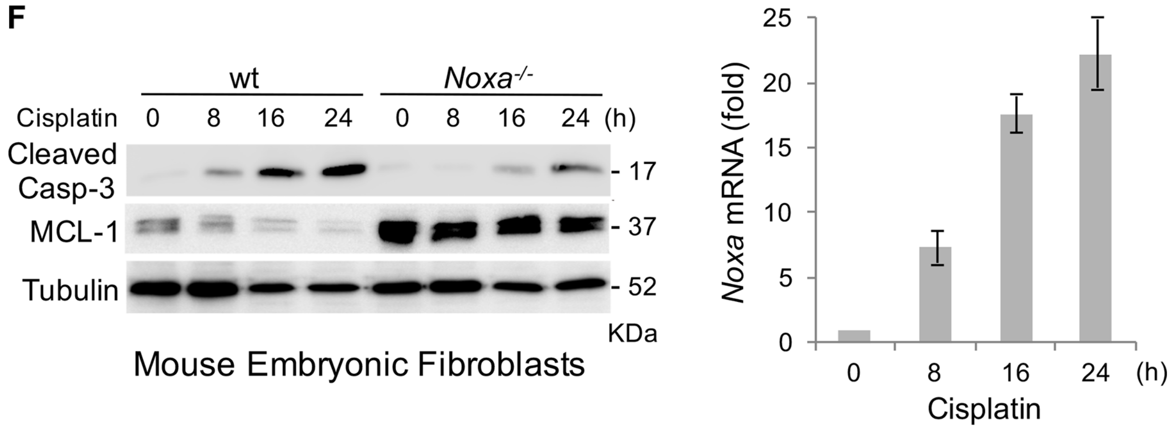


**Supplementary Figure S1: Noxa is required for DNA damaging agents-induced MCL-1 degradation and apoptosis in HeLa cervical, MDA-MB-468 breast cancer, HN30 HNSCC, and mouse embryonic fibroblasts (MEFs).** (**A**) HeLa cells were infected with lentivirus encoding shRNA for non-targeting control or Noxa. Cells were then treated with etoposide (50 μM) for the indicated periods and equal amounts of total extracts were subjected to immunoblot analysis using the indicated antibodies. (**B**) HeLa cells in (A) were treated with etoposide for 24 h and cell death was determined by Annexin V-PI staining followed by FACS analyses. (**C**) HeLa cells were infected with retrovirus encoding shRNA for non-targeting control, Noxa #2, or Noxa #3. Cells were treated with cisplatin (30 μM) or etoposide (50 μM) for 24 h and cell death was determined by a trypan-blue exclusion assay. (Left panel). The expression of Noxa was determined by immunoblot analysis (Right panel). (**D**) MDA-MB-468 breast cancer cells were infected with lentivirus encoding shRNA for non-targeting control or Noxa. Cells were then treated with cisplatin (10 μM) or etoposide (50 μM) for the indicated periods and equal amounts of total extracts were subjected to immunoblot analysis using the indicated antibodies. (**E**) HN30 HNSCC cells were infected with lentivirus encoding shRNA for non-targeting control or Noxa. Cells were then treated with cisplatin (20 μM) for the indicated periods and equal amounts of total extracts were subjected to immunoblot analysis using the indicated antibodies. (**F**) Wild-type (Wt) or Noxa-/- MEFs were treated with cisplatin (30 μM) for the indicated periods and equal amounts of total extracts were subjected to immunoblot analysis using the indicated antibodies (Left panel). The levels of Noxa mRNA in wt MEFs was determined by qRT-PCR and was normalized with the value of β-Actin mRNA (Right panel).

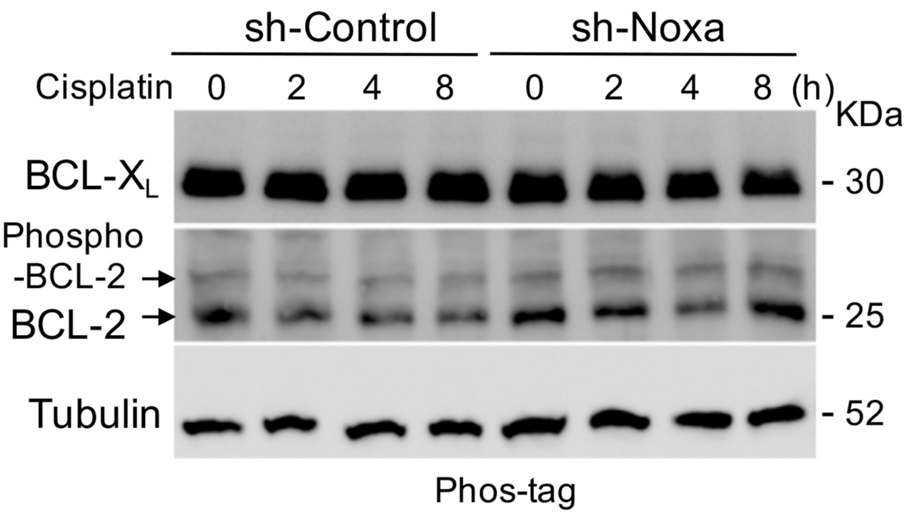


**Supplementary Figure S2: The phosphorylation statuses of BCL-2 and BCL-$X_L$ are not changed by cisplatin treatment.** HeLa cells were infected with lentivirus-encoding shRNA for non-targeting control or Noxa. Cells were then treated with cisplatin (30 μM) for the indicated periods and equal amounts of total extracts were applied in SDS-PAGE with 30 μM Phos-tag.

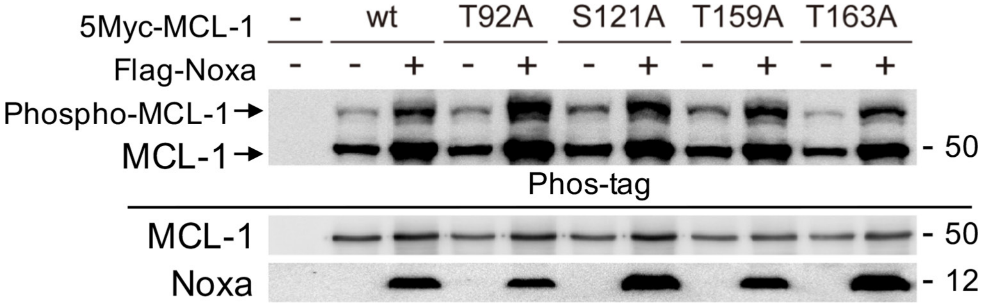


**Supplementary Figure S3: Substitution from Thr92, Ser121, Thr159, or Thr163 to Ala does not affect the phosphorylation of MCL-1 by Noxa expression.** Myc-tagged human MCL-1 wild-type (wt) or the mutants in which Ser and/ or Thr were substituted to Ala were co-transfected with human Noxa in 293T cells for 24 h. Equal amounts of total extracts were subjected to immunoblot analysis using the indicated antibodies.

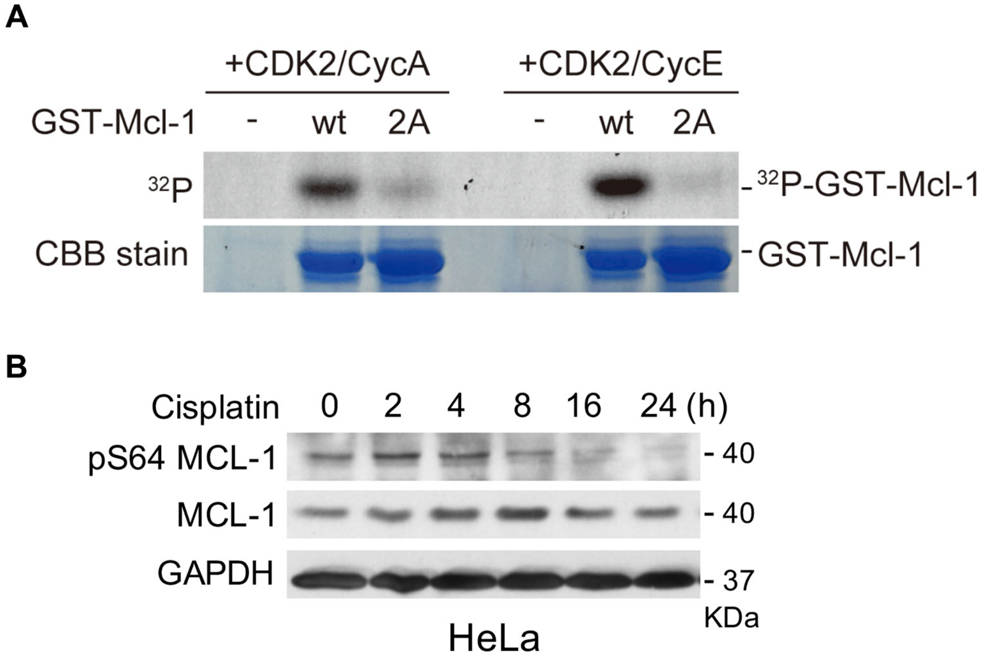


**Supplementary Figure S4: Ser64 and Thr70 can be directly phosphorylated by CDK2 and is phosphorylated by cisplatin treatment.** (**A**) Purified GST-MCL-1 (wt or 2A) and the active CDK2 (CDK2/Cyclin A or CDK2/Cyclin E) were incubated with $^{32}\gamma$-ATP at 30°C for 30 min. The samples were resolved by SDS-PAGE and phosphorylated GST-MCL-1 was detected by autoradiography. (**B**) HeLa cells were treated with cisplatin (30 μM) for the indicated periods and equal amounts of total extracts were subjected to immunoblot analysis using the indicated antibodies.

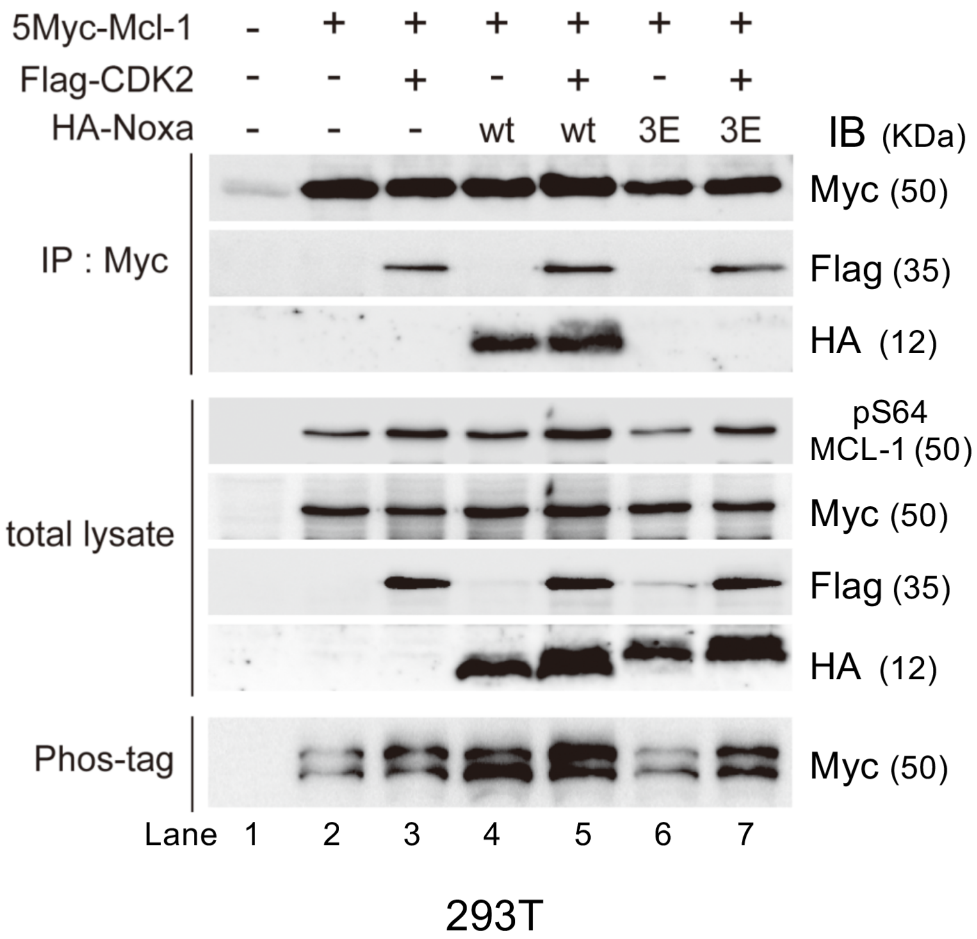


**Supplementary Figure S5: A Noxa BH3 mutant can neither bind to MCL-1 nor enhance MCL-1 phosphorylation.** HA-tagged Noxa (wt or 3E), Myc-tagged MCL-1, and/or Flag-tagged CDK2 were transfected into 293T cells for 24 h. Total extracts were immunoprecipitated with anti-Myc antibodies followed by immunoblotting with the indicated antibodies to detect the molecular interactions. Phosphorylation of MCL-1 was detected with phospho-Ser64 specific antibodies or by SDS-PAGE with Phos-tag.
